# The mechanism of dehydroandrographolide inhibiting metastasis in gastric cancer based on network pharmacology and bioinformatics

**DOI:** 10.1097/MD.0000000000034722

**Published:** 2023-08-25

**Authors:** Yan-hai Luo, Ling Yuan, Dou-dou Lu, Ya-ting Yang, Yi Yang, Yu-hua Du, Jun-fei Zhang, Yan Chen, Lei Zhang, Yi Nan

**Affiliations:** a Pathology of Department, General Hospital of Ningxia Medical University, Yinchuan, China; b Pharmacy College of Ningxia Medical University, Yinchuan, China; c Key Laboratory of Hui Ethnic Medicine Modernization of Ministry of Education, Ningxia Medical University, Yinchuan, China; d School of Clinical Medicine, Ningxia Medical University, Yinchuan, China.

**Keywords:** bioinformatics, dehydroandrographolide, gastric cancer, metastasis, molecular docking

## Abstract

Gastric cancer (GC) is the most aggressive malignant tumor of the digestive tract. However, there is still a lack of effective treatment methods in clinical practice. Studies have shown that dehydroandrographolide (DA) has been shown to have anti-cancer activity in a variety of cancers, but it has not been reported in GC. Firstly, we obtained data on DA target genes, GC-related genes, and differentially expressed genes (DEGs) from the PharmMapper, GeneCards, and GEO databases, respectively. Then, the STRING database was used to construct the protein–protein interaction network of intersection genes, and Gene Ontology and Kyoto Encyclopedia of Genes and Genomes analyses of intersection genes were performed. Finally, 8 hub target genes were identified by analyzing their expression and prognostic survival, and molecular docking between the hub genes and DA was performed. In this study, 293 DA drug target genes, 11,366 GC-related genes, and 3184 DEGs were identified. Gene Ontology and KEGG analysis showed that the intersection genes of DA targets and GC-related genes were mainly related to cancer pathways involving apoptosis and cell adhesion. The intersection genes of DEGs, DA targets, and GC-related genes were also mainly related to cancer pathways involving chemical carcinogenesis, and drug metabolism. The molecular docking results showed that the 8 hub target genes had an apparent affinity for DA, which could be used as potential targets for DA treatment of GC. The results of this study show that the molecular mechanism by which DA inhibits GC metastasis involves multiple target genes. It may play an essential role in inhibiting the invasion and metastasis of GC by regulating the expression and polymorphism of hub target genes, such as *MMP9, MMP12, CTSB, ESRRG, GSTA1, ADHIC, CA2*, and *AKR1C2*.

## 1. Introduction

Gastric cancer (GC) is one of the most common malignant tumors of the digestive tract. Its incidence ranks fourth among cancers worldwide, and its mortality ranks third.^[1]^ According to the analysis data of the International Agency for Research on Cancer, there were approximately 770,000 deaths from GC worldwide in 2020, of which China accounted for approximately 48%, which was significantly higher than that of other countries.^[2]^ At present, the treatment of GC is mainly based on surgery, radiotherapy, and chemotherapy. However, surgical treatment is ineffective for metastatic patients, and chemotherapy drugs, such as cisplatin, and 5-fluorouracil have side effects; long-term use not only leads to reduced patient compliance but also leads to serious drug resistance.^[3]^ Some natural traditional Chinese medicine ingredients have been proven to have anti-tumor activity and are often used as adjuvants for chemotherapy drugs. When taken together with chemotherapy drugs, they can improve the sensitivity of patients to chemotherapy drugs and reduce the side effects.^[4,5]^

Dehydroandrographolide is a diterpene lactone compound extracted from andrographis. Numerous studies have shown that dehydroandrographolide (DA) has anti-inflammatory,^[6,7]^ anti-allergy,^[8,9]^ antiviral,^[10]^ hepatobiliary protection,^[11]^ and other drug activities. In recent years, it has been found that DA shows effective anti-cancer activity in various cancers, mainly by inhibiting proliferation, promoting apoptosis, and reducing the migration and invasion of cancer cells. In osteosarcoma, DA can inhibit osteosarcoma cell growth and metastasis by reversing the epithelial to mesenchymal transition (EMT) by targeting SATB2.^[12]^ In oral cancer, DA can inhibit the invasion and metastasis of SCC9 cells by inhibiting the expression of MMP2 and the EMT process, and be used as a potential chemopreventive agent against oral cancer metastasis.^[13]^ As a TMEM16A inhibitor in colon cancer, DA could significantly inhibit the invasion and metastasis of SW620 cells.^[14]^ In GC, no relevant literature has been reported; therefore, this study has potential research significance. This study used network pharmacology and bioinformatics methods to explore the potential molecular mechanism by which DA inhibits the progression and provides a the theoretical basis for its use as a clinical treatment drug for GC.

The continuous deterioration of tumors is mainly caused by cancer cell invasion and metastasis, it is also the leading cause of tumor treatment failure and death. Tumor metastasis can be divided into 3 categories: direct spread, lymphatic metastasis, and hematogenous metastasis. Among them, lymphatic metastasis and hematogenous metastasis are the most common modes of metastasis, and the process can be summarized in 3 stages. The first stage is intravasation, in which tumor cells break through the basement membrane and enter the lymphatic and blood circulation systems. The second stage is extravasation, in which tumor cells break through the blood vessels and invade other organs. The third stage is tumor angiogenesis, in which tumor cells grow in secondary tissues and organs and accompany tumor angiogenesis.^[15]^ Tumor metastasis involves multiple biological processes, such as immune escape, dynamic changes in the basement membrane, tumor angiogenesis, and EMT.^[16]^ Among them, EMT is essential in mediating tumor metastasis by regulating the expression of extracellular matrix metalloproteinases (MMPs), the integrin family, and transforming growth factors.^[17]^ In this study, through the analysis of the intersection genes of DEGs, DA targets, and GC-related genes (DA-GC-DEGs), we found that the intersection genes were mainly closely related to intercellular adhesion, adhesive junctions, integrin complex, transforming growth factor ligand and receptor complexes, etc. The above events indicated that DA-GC-DEGs may be related to EMT, and we speculate that DA is likely to inhibit the invasion and metastasis of GC cells through the regulation of EMT.

## 2. Materials and methods

### 2.1. Acquisition of DA target genes and GC-related genes

Firstly, the 3D structure of DA was obtained from the PubChem website (https://pubchem.ncbi.nlm.nih.gov/), then the 3D structure was submitted on the PharmMapper website (http://www.lilab-ecust.cn/pharmmapper/) to obtain the DA target genes. Finally, the VLOOKUP function was used to compare the DA target genes with human gene targets obtained from the Uniprot database (https://www.uniprot.org/), and 293 of the 300 target genes were retained. GC-related genes were obtained from the GeneCards database (https://www.genecards.org/), with “Gastric cancer” as the search term and 11,366 GC-related genes were retrieved, and each gene contained information such as gene ID, correlation score, and link to the website in GeneCards. Among GC-related genes, genes with correlation scores ≥ 10 (891) were intersected with DA target genes (293) to obtain DA-GC, and all genes (11,366) were intersected with DA target genes and DEGs (1818) to obtain DA-GC-DEGs.

### 2.2. Acquisition of GEO dataset and differentially expressed genes analysis

The GC microarray dataset was obtained from the GEO website (https://www.ncbi.nlm.nih.gov/geo/). The dataset numbered GSE118916 was downloaded, which has a total of 30 samples, including 15 pairs of GC tissue and adjacent non-tumor tissues. Finally, all samples were divided into a tumor and a normal group. Then, using logFC and *P* values as a filter condition, genes with |LogFC| ≥1 and *P* < .05 were considered as significantly differentially expressed genes (DEGs). A total of 3184 DEGs were obtained, and 1818 genes were retained after removing duplicate genes.

### 2.3. Protein–protein interaction and network topology analysis

The bioinformatics online (http://www.bioinformatics.com.cn/) was used to identify intersection targets between GC and DA, and the intersection genes were imported into the String database (https://cn.string-db.org/) to obtain the information on the protein interaction network. Cytoscape 3.9.1 software was used to construct the protein–protein interaction (PPI) network of intersection genes according to the degree size and to show the degree values of the top 25 genes.

### 2.4. Gene Ontology analysis and Kyoto Encyclopedia of Genes and Genomes pathway enrichment analyses

The intersection genes of DA-GC and DA-GC-DEGs were annotated and clustered using the Metascape database (https://metascape.org/), and the above results were visualized by bioinformatics online platform (http://www.bioinformatics.com.cn) for Gene Ontology (GO) analysis and Kyoto Encyclopedia of Genes and Genomes (KEGG) pathway enrichment analyses. GO analysis included 3 types: biological process (BP), cellular component (CC), and molecular function (MF). The top 10 GO analysis results and the top 20 KEGG pathway enrichment results are further shown in the form of an enrichment dot bubble.

### 2.5. Expression and survival analysis for hub genes

The 5 most significantly up-regulated and down-regulated genes were selected as hub genes from GC-DA-DEGs. With |LogFC| ≥1 and *P* < .05 as the screening conditions, the GEPIA database (http://gepia.cancer-pku.cn/) was used to analyze the expression of hub genes in GC and normal gastric tissues. The Kaplan–Meier Plotter database (https://kmplot.com/) was used to analyze the correlation between the expression and overall survival of hub genes in GC. With log-rank *R* ≤ 0.01 as the screening condition, genes with significant differences were screened for molecular docking verification.

### 2.6. Molecular docking

The 2D structure of DA was obtained from the PubChem database (https://pubchem.ncbi.nlm.nih.gov/), then the 2D structure was imported into Chem3D software to get the 3D structure and saved as PDBQT by the AutoDockTools software (http://autodock.scripps.edu/). The protein crystal structure with high resolution was selected as the acceptor, and its PDB format was downloaded from the RCSB PDB database (https://www.rcsb.org/), imported with PYMOL software to remove water molecules, and then imported with AutoDockTools software to add hydrogen atoms and saved in PDBQT format. Finally, with the drug structure as the ligand and the protein structure as the receptor, a docking box was formed using AutoDockTools software, and the results with the lowest docking energy were saved. The result with the lowest docking energy was visualized using PYMOL software, and the name of the amino acid residue and the size of the hydrogen bond in the docking site were noted.

## 3. Results

**Figure 1.**
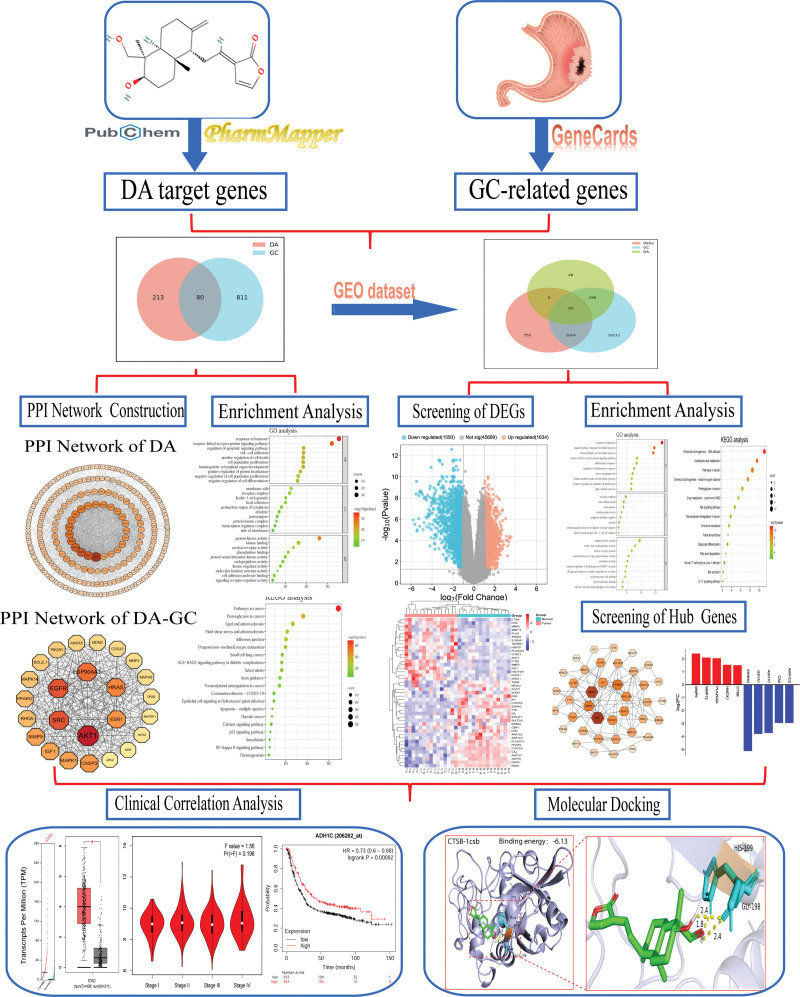
The workflow of DA inhibiting metastasis of gastric cancer. Arrows in the figure express the order of the analysis procedure. DA = dehydroandrographolide, DA-GC = the intersection genes of DA targets genes and GC-related genes, DEGs = differentially expressed genes, GC = gastric cancer, PPI = protein–protein interaction.

This study was divided into 3 phases. In the first stage, DA target genes and GC-related genes were obtained, and the intersection genes were screened by Venn diagram, and then GO analysis and KEGG pathway enrichment analyses were performed on the intersection genes. In order to further narrow the scope of core target genes, we introduced GC microarray data from the GEO database in the second stage and used a Venn diagram to obtain the DA-GC-DEGs. Similarly, GO and KEGG pathway enrichment analyses were performed on the intersection genes. In the third stage, the expression and survival prognosis of the ten hub genes were analyzed, and 8 genes with significant differences were further screened for molecular docking verification. The workflow is shown in Figure 1.

### 3.1. Acquisition of target genes and construction of PPI network

**Figure 2.**
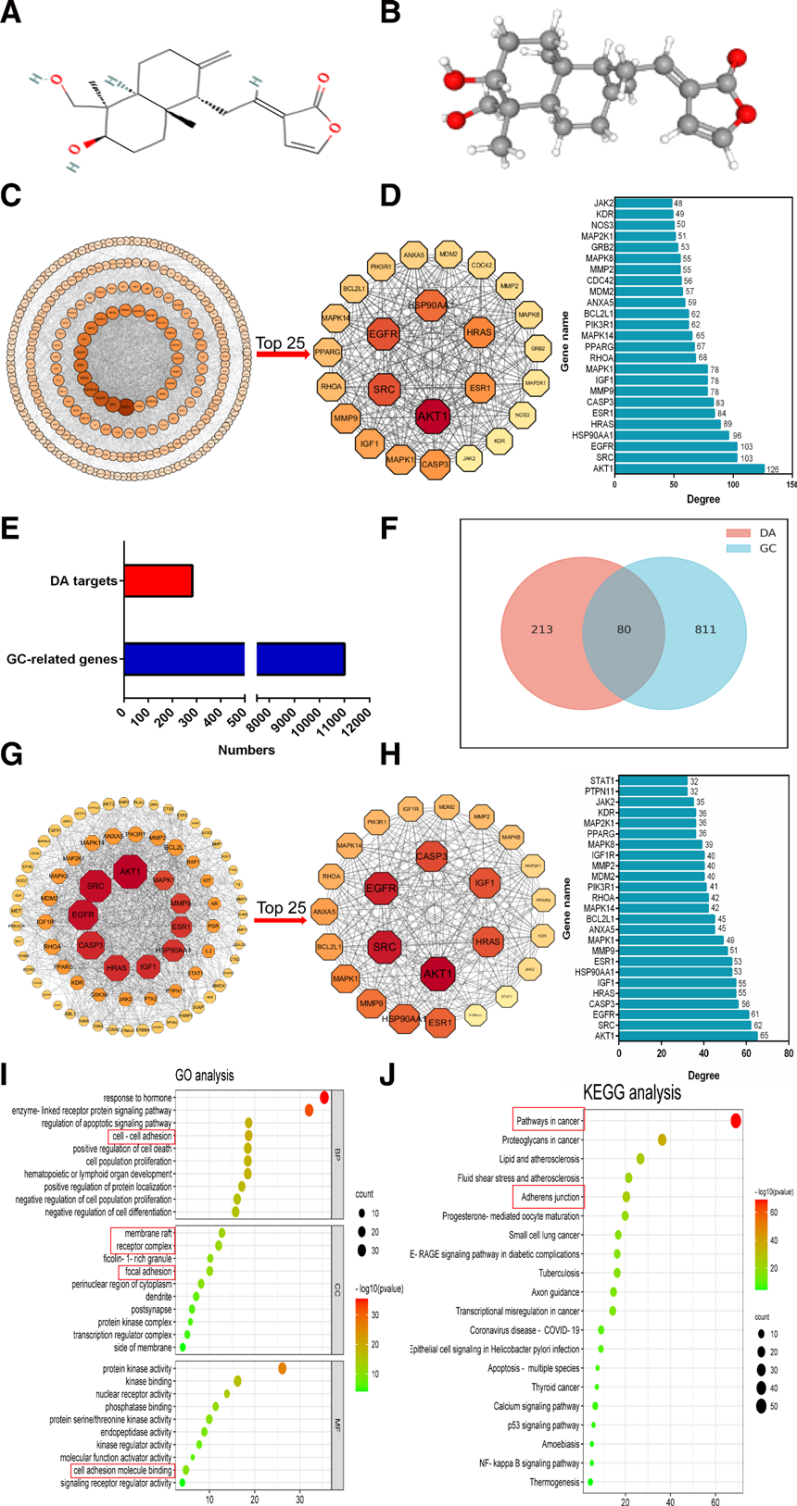
PPI network construction and functional analysis of DA-GC. (A) 2D structural diagram of DA. (B) 3D structure diagram of DA. (C) PPI network of DA target genes, the larger the circle and the color close to red, the greater the degree value (D) The PPI network and corresponding degree values of the top 25 DA target genes. (E) DA and GC-related genes. (F) Intersection of DA target gene and GC-related genes. (G) The PPI network of DA-GC. (H) The PPI network and corresponding degree values of the top 25 intersection genes. (I and J) GO and KEGG pathway enrichment analyses of DA-GC, the bubble size represents the proportion of genes on each biological process, the larger the bubble, the more genes are enriched, the color represents the degree of enrichment, and the closer the color is to red, the more significant the enrichment. Red boxes represent functions and pathways associated with cancer and metastasis. DA = dehydroandrographolide, DA-GC = the intersection genes of DA targets genes and GC-related genes, GC = gastric cancer, GO = Gene Ontology, PPI = protein–protein interaction, KEGG = Kyoto Encyclopedia of Genes and Genomes.

**Figure 3.**
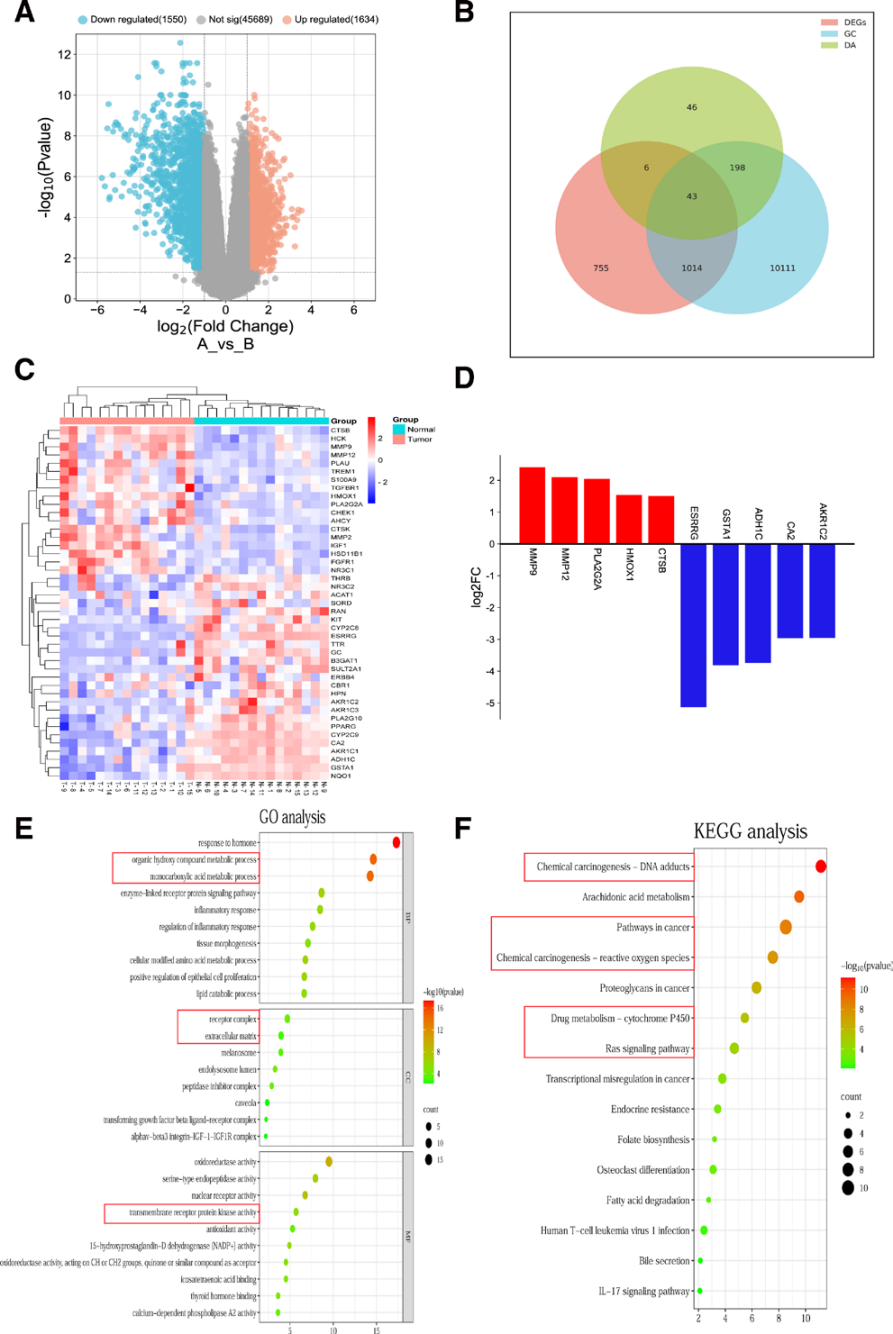
Acquisition of DA-GC-DEGs and identification of hub genes. (A) The volcano plot of DEGs. Red represents up-regulated genes, blue represents down-regulated genes, and gray represents no difference in genes. (B) The Venn diagram of DA-GC-DEGs. (C) Heat maps of DA-GC-DEGs, the closer the color is to red, the more expressive it is. (D) Hub genes, red represents up-regulated genes while green represents down-regulated genes. (E and F) GO and KEGG pathway enrichment analyses of DA-GC-DEGs, the bubble size represents the proportion of genes on each biological process, the larger the bubble, the more genes are enriched, the color represents the degree of enrichment, and the closer the color is to red, the more significant the enrichment. Red boxes represent functions and pathways associated with cancer and metastasis. DA-GC = the intersection genes of DA targets genes and GC-related genes, DEGs = differentially expressed genes, GO = Gene Ontology, KEGG = the Kyoto Encyclopedia of Genes and Genomes.

The 2D (Fig. 2A) and 3D structures (Fig. 2B) of DA were obtained from the PubChem website. A total of 300 DA target genes were obtained from the PharmMapper website, and after comparing the DA target genes with the human gene targets by the VLOOKUP function, 293 genes were retained (Fig. 2E). Subsequently, Cytoscape software was used to construct the PPI network among the target genes (Fig. 2C), and the top 25 target genes and corresponding degree values are shown in Figure 2D. In total, 11,366 GC-related genes were obtained from the GeneCards database (Fig. 2E). Genes with a correlation score ≥ 10 were intersected with DA target genes, and a total of 80 intersecting genes were obtained (Fig. 2F). The PPI network of DA-GC was constructed using Cytoscape software (Fig. 2G). The top 25 target genes and their corresponding degrees are shown in Figure 2H. Through comparison, we found that the DA target genes ranked in the top 25 were highly similar to the DA-GC ranked in the top 25, and 22 of the 25 genes were consistent, except for CDC42, GRB2, and NOS3.

### 3.2. GO and KEGG analysis of DA-GC

GO analysis showed that in BPs, the intersection genes were mainly concentrated in response to hormones, enzyme-linked receptor protein signaling pathways, cell-cell adhesion, positive regulation of cell death, cell population proliferation, and negative regulation of cell differentiation, etc. In CCs, the intersection genes were mainly concentrated in the membrane raft, receptor complex, focal adhesion, dendrite, protein kinase complex, and transcription regulator complex. In MFs, the intersection genes mainly focused on protein kinase activity, nuclear receptor activity, phosphatase binding, and cell adhesion molecules binding (Fig. 2I). KEGG pathway enrichment analyses showed that intersection genes were mainly concentrated in cancer-related signaling pathways, lipids and atherosclerosis, adherens junction, cytoskeleton regulation, cancer transcriptional irregulation in cancer, and the P53 signaling pathway (Fig. 2J). The above events suggest that DA may be related to the invasion and metastasis of GC, but the mechanism needs to be further explored.

### 3.3. Screening of hub genes

Based on the analysis of the GC microarray data set obtained from the GEO database, a total of 3184 DEGs were identified, including 1634 up-regulated genes and 1550 down-regulated genes (Fig. 3A). In order to further screen the core target genes, we obtained the DA-GC-DEGs by using a Venn diagram. A total of 43 intersection genes were obtained (Fig. 3B), of which 18 genes were up-regulated and 25 genes were down-regulated in GC (Fig. 3C). From the intersection genes, the top 5 up-regulated and top 5 down-regulated genes were selected as hub genes; the up-regulated genes were *MMP9, MMP12, PLA2G2A, HMOX1*, and *CTSB*; the down-regulated genes were *ESRRG, GSTA1, ADH1C, CA2*, and *AKR1C2* (Fig. 3D).

### 3.4. GO and KEGG analysis of DA-GC-DEGs

By drawing the Venn diagram of DA-GC-DEGs, 43 intersection genes were screened, and GO and KEGG pathway enrichment analyses were performed on the intersection genes. GO analysis showed that in BPs, the intersection genes were mainly concentrated in response to hormones, organic hydroxy compound metabolic processes, enzyme-linked receptor protein signaling pathways, inflammatory responses. In terms of CCs, the intersection genes were mainly receptor complexes, extracellular matrix, caveolae, transforming growth factor beta ligand-receptor complex, and ITGAV-ITGβ3-IGF1-IGF1R complex. In terms of MFs, the intersection genes were mainly related to oxidoreductase activity, serine-type endopeptidase activity, nuclear receptor activity, and transmembrane receptor protein kinase activity (Fig. 3E). KEGG pathway enrichment analyses showed that the intersection genes were mainly related to chemical carcinogenesis, arachidonic acid metabolism, pathways in cancer, the Ras signaling pathway, and transcriptional irregulation in cancer (Fig. 3F).

### 3.5. Clinical correlation analysis of hub genes

**Figure 4.**
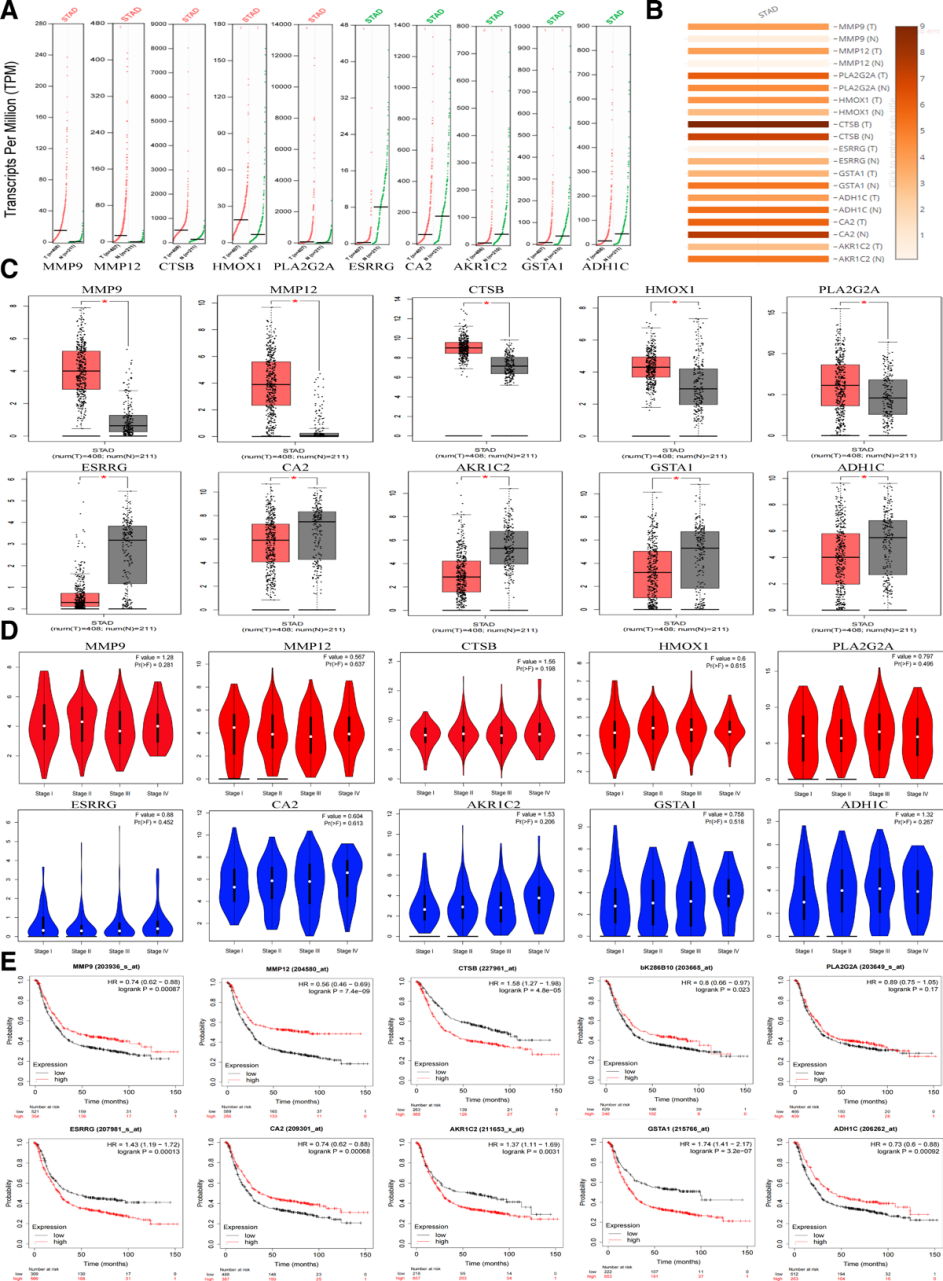
Analysis of hub genes expression and survival. (A–D) GEPIA analysis of hub genes, the results were based on data from the GEPIA database (http://gepia.cancer-pku.cn/). (A) The TPM values of the hub genes. The tumor group contained 408 samples and the normal group contained 211 samples. (B and C) Expression of hub gene in gastric cancer and normal gastric tissues. The tumor group contained 408 samples and the normal group contained 211 samples. (D) Individual cancer stage of hub genes in gastric cancer. Each subplot contains 619 samples. (E) Survival analyses for hub genes, the results were based on data from the public database KMplot (www.kmplot.com). Each subplot contains 817 samples. TPM = transcripts per million.

Based on the GEPIA database, we analyzed the expression of ten hub genes in GC and normal gastric tissues. The results showed that the transcripts per million values of up-regulated genes such as *MMP9, MMP12, PLA2G2A, HMOX1*, and *CTSB* in GC were significantly higher than in normal gastric tissues. On the contrary, the transcripts per million values of down-regulated genes such as *ESRRG, GSTA1, ADH1C, CA2*, and *AKR1C2* in GC tissues showed a significant downward trend (Fig. 4A), and similar differences were observed in protein expression (Fig. 4B and C). However, in terms of clinical stage, there were no significant differences in hub genes (Fig. 4D). We used the Kaplan–Meier Plotter database to analyze the relationship between protein expression levels and the survival of hub genes. The results showed that the expression of *MMP9, MMP12, CA2, ADH1C, CTSB, ESRRG, AKR1C2*, and *GSTA1* were correlated with survival prognosis. Neither *HMOX1* nor *PLA2G2A* expression levels were significantly associated with survival prognosis (Fig. 4E). It should be noted that the relationship between *MMP9, MMP12*, and *ESRRG* expression and prognosis may not be in line with the actual situation. *MMP9* and *MMP12* are members of the matrix metalloprotein, and the higher their expression, the worse the prognosis of patients. ESRRG may act as a tumor suppressor in GC, and the higher its expression, the better the prognosis and survival of patients.

### 3.6. Molecular docking of DA and hub genes

**Figure 5.**
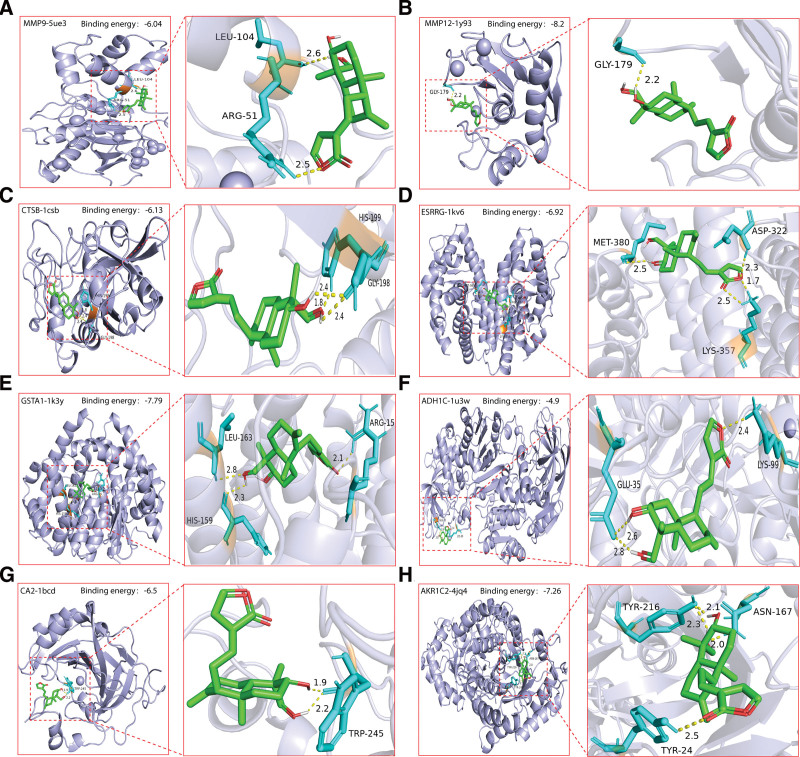
Molecular docking between hub genes and DA. (A) MMP9. (B) MMP12. (C) CTSB. (D) ESRRG. (E) GSTA1. (F) ADH1C. (G) CA2. (H) AKR1C2. Bright blue is the protein, green is the drug, orange is the amino acid linked to the drug, cyan is the amino acids stick structure, yellow dashed lines are hydrogen bonds, and numbers are bond lengths. DA = dehydroandrographolide.

By analyzing the expression and survival prognosis of hub genes in GC, we selected 8 proteins with significant statistical differences for molecular docking. The protein crystals corresponding to the hub genes were searched using the PDB database, and the protein crystal names corresponding to MMP9, MMP12, CTSB, ESRRG, GSTA1, ADH1C, CA2, and AKR1C2 were 5ue3, 1y93, 1csb, 1kv6, 1k3y, 1u3w, 1bcd, and 4jq4, respectively. The molecular docking results are shown in Figure 5. It is generally believed that a lower docking energy indicates a better docking effect. If the docking energy is less than 0 kcal/mol, it indicates that the drug and protein can combine under natural conditions, and if the docking energy is less than −5 kCal/mol, the binding effect is excellent. In this study, MMP12 and GSTA1 had the best docking efficiency with docking energy of −8.2 and −7.79 kCal/mol, respectively, followed by AKR1C2 and ESRRG with docking energy of −7.26 and −6.92 kcal/mol, respectively. ADH1C and MMP9 showed the worst docking results with docking energies of −4.9 and −6.04 kCal/mol, respectively, followed by CTSB and CA2 with docking energies of −6.13 and −6.5 kCal/mol, respectively.

## 4. Discussion

GC is a common malignant digestive tract tumor with high mortality, poor prognosis, easy recurrence, and easy metastasis. In this study, based on network pharmacology and bioinformatics techniques, we found that DA may be involved in the regulation of GC invasion and metastasis.

Firstly, we obtained DA target genes and GC-related genes and then performed GO and KEGG analysis of their intersection genes, which showed that the intersection genes were mainly related to cell-cell adhesion, focal adhesion, transcription regulator complex, and adhesion junction. These events suggest that DA may be associated with the invasion and metastasis of GC.

Subsequently, to identify core target genes, we added GC microarray data to obtain DA-GC-DEGs and performed GO and KEGG analyses, which showed that the DA-GC-DEGs were also mainly related to cancer pathways involving chemical carcinogenesis, the Ras signaling pathway, integrins, and the extracellular matrix. Similarly, the results suggested that DA may be related to the invasion and metastasis of GC.

Finally, the top 5 up-regulated and top 5 down-regulated genes in DA-GC-DEGs were selected as hub genes. The up-regulated genes were *MMP9, MMP12, PLA2G2A, HMOX1*, and *CTSB*, and the down-regulated genes were *ESRRG, GSTA1, ADH1C, CA2*, and *AKR1C2*. The expression and prognosis of hub genes in GC were analyzed using the GEPIA and the Kaplan–Meier Plotter databases, respectively. Eight hub genes with statistically significant differences were selected for molecular docking analysis.

Through the analysis of hub genes, we found that MMP9 could be used as a direct target for multiple drugs in GC. By reducing the expression of MMP9 and other metastasis-related proteins, such as S100A4, CD147, and TGFB1, EMT and tumor angiogenesis are inhibited, and tumor invasion and metastasis activities are also decreased.^[18–20]^ MMP12 and other members of the MMP family, such as MMP1, MMP3, MMP7, MMP9, and MMP15, are involved in regulating the invasion and metastasis of GC.^[21–23]^ In addition, the high expression of MMP12 in GC is closely related to tumor invasion, lymphatic metastasis, and TNM staging, which can be used as an independent indicator for the diagnosis and prognostic analysis of GC.^[24]^ CTSB is highly expressed in lung cancer, breast cancer, colon cancer, prostate cancer, and other tumor tissues, especially in GC. The CTSB gene polymorphism can be used as an important indicator to predict the risk and prognosis of GC.^[25]^ As a tumor suppressor, ESRRG can inhibit the proliferation of GC cells induced by Helicobacter pylori infection.^[26]^ As a negative regulator of the Wnt signaling pathway, ESRRG can inhibit the occurrence and development of GC by inhibiting the binding of TCF4/LEF1 and CCND1 promoter,^[27]^ and also inhibit the proliferation, migration, and invasion of GC cells by promoting the degradation of β-catenin.^[28]^ GSTA1 is expressed at low levels in GC and its gene polymorphism are closely related to the occurrence of GC.^[29,30]^ It can be used as a prognostic marker and targeted biomarker for gastric adenocarcinoma after chemotherapy.^[31]^ ADH1C gene polymorphism are highly correlated with the risk of GC.^[32]^ AKR1C2 can regulate the differentiation, proliferation and metastasis of GC cells through the twist signaling pathway, and it is a potential target gene for the treatment of GC.^[33,34]^

In conclusion, we suggest that DA treatment of GC may be related to the inhibition of invasion and metastasis. DA may inhibit the progression of GC by regulating the expression or gene polymorphism of hub genes.

## 5. Conclusion

**Figure 6.**
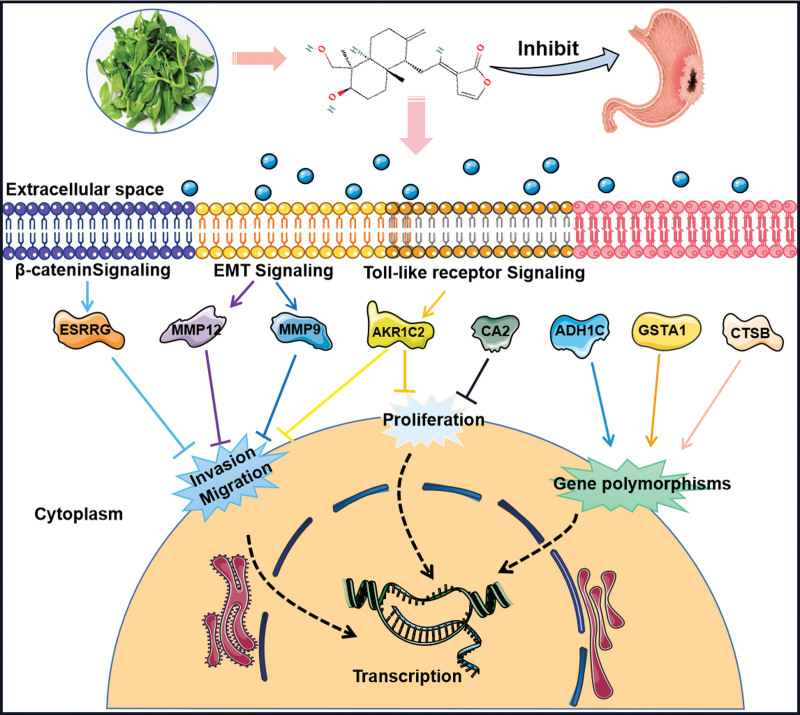
Schematic drawing of the mechanism of DA in the treatment of gastric cancer.

In this study, the potential function of DA in GC treatment was identified based on network pharmacology and molecular docking technology. The results showed that DA might be involved in regulating the invasion and metastasis of GC by regulating the expressions of MMP9, MMP12, ESRRG, and gene polymorphisms of *ADH1C, GSTA1*, and *CTSB*. A possible mechanism is shown in Figure 6. In future studies, we will verify these conclusions further.

## Acknowledgments

We sincerely thank the public databases mentioned in this article for generously sharing a large amount of data. We also thank Ms. Joanna for reviewing and polishing the manuscript.

## Author contributions

**Data curation:** Yan-hai Luo, Dou-dou Lu.

**Funding acquisition:** Ling Yuan, Jun-fei Zhang, Yi Nan.

**Methodology:** Yan-hai Luo, Yu-hua Du.

**Resources:** Yan-hai Luo, Ya-ting Yang.

**Software:** Yan-hai Luo, Dou-dou Lu.

**Supervision:** Ling Yuan, Yi Nan.

**Visualization:** Yan-hai Luo, Yi Yang.

**Writing – original draft:** Yan-hai Luo, Yan Chen.

**Writing – review & editing:** Jun-fei Zhang, Lei Zhang, Yi Nan.
